# Preventive and promotive effects of habitual hot spa-bathing on the elderly in Japan

**DOI:** 10.1038/s41598-017-18488-3

**Published:** 2018-01-09

**Authors:** Toyoki Maeda, Koshi Mimori, Sadao Suzuki, Takahiko Horiuchi, Naoki Makino

**Affiliations:** 1 The Department of Internal Medicine, Kyushu University Beppu Hospital, 4546 Tsurumihara, Beppu, Oita, 874-0838 Japan; 2 The Department of Surgery, Kyushu University Beppu Hospital, 4546 Tsurumihara, Beppu, Oita, 874-0838 Japan; 3 Department of Preventive Medicine, Nagoya City University Graduate School of Medical Sciences, 1 Kawasumi, Mizuho-cho, Mizuho-ku, Nagoya, 467-8601 Japan

## Abstract

Although body-warming with hot spa-bathing has been proposed to exert medical therapeutic effects on certain diseases, whether body-warming has preventive and promotive effects remains unknown. To clarify this issue, an epidemiological questionnaire study regarding personal hot spa-bathing habits and disease history was carried out in Japan, where individuals engage in daily warm water bathing. Questionnaires regarding hot spa-bathing habits and disease history were randomly sent to 20,000 residents aged ≥65 years living in Beppu, a city in Japan that has the highest concentration of hot spa sources in the world. The results showed that habitual hot spa-bathing exerts preventive or promotive effects on the occurrence of certain diseases, such as hypertension (preventive) and collagen disease (promotive) in women, and cardiovascular diseases (preventive) and colon cancer survival (promotive) in men. These findings suggest that habitual body warming is an effective and economical method with beneficial preventive and promotive effects on various diseases.

## Introduction

Does habitual body-warming lead to any beneficial medical effects in humans? Although this is a simple question, little remains known about the effects of systemic-heating on the human body, including whether habitual body warming is beneficial or harmful for health. In Western countries, body-warming activities are not habitual, although there are numerous hot spa resorts in Europe, where balneotherapy is covered by national health insurance as medical treatment; however, the duration of balneotherapy is limited to within several weeks. Therefore, the biological effects of long-term balneotherapy remain unknown. In addition, balneotherapy is only prescribed for patients, not for healthy people.

In Japan, people have a long history of habitual daily bathing that involves warm water bathing as opposed to showering. They typically use warm water from their own homes for bathing, but in hot springs areas, which are prevalent in regions all over Japan, people prefer to take habitual hot spa baths, which contain rich minerals. Compared with home bathing, hot spa-bathing in water rich in minerals that foster heat-conduction is more effective for raising core body temperature. The Japanese Ministry of the Environment has published a list of the balneotherapeutic effects of spa-bathing with respect to hot spa type^1^. According to a list of minerals found in hot spas between 1978 and 2014, spa water quality can be categorized in to 11 distinct types. This list shows the association between each spa type and its target diseases. However, the prophylactic effect of spa-bathing remains unknown. Kyushu University Beppu Hospital is located in Beppu city, which contains the world highest concentration of hot spa sources. This area has nine of the 11 spa types. Thus, Beppu city is one of best areas to conduct epidemiological studies about the disease preventive effects of habitual hot spa-bathing. Therefore, we conducted an epidemiological questionnaire study on elderly residents of Beppu city regarding personal hot spa-bathing habits and disease history.

## Results

The profiles and bathing habits of the respondents are shown in Tables 1–3. For reliability, we included only respondents using three spa types (simple, chloride, and bicarbonate), each of which contained more than 100 bathers.

**Table 1:** Age distribution and frequency of hot spa-bathing among the respondents.

	Total	Men		Women		Unknown
Received	11146	4706		6352		88
Valid	9252	4081		5171		0
**Age (years)**	**Total**	**Men**		**Women**		
65–69	2920	1292		1628		
70–74	2588	1126		1462		
75–79	2158	965		1193		
80–84	1064	493		571		
≥85	522	205		317		
**Spa bathing frequency**	**Total**	**Men**		**Women**		
<1/month = non-spa bathers	2322	990		1332		
1/month–1/week	330	170		160		
2–3/week	781	372		409		
4–5/week	1271	563		708		
Daily	4548	1986		2562		
**Age (years)**	**65–69**	**70–74**	**75–79**	**80–84**	**≥85**	**Total**
**Non-spa bathers**	831	607	517	241	126	2322
**Spa bathers**	2089	1981	1641	823	396	6930

The following data with regard to bathing conditions are from those who engaged in hot

spa-bathing once a month or more.

**Table 2:** The respondents’ bathing conditions.

	Total	Men	Women
Duration of immersion in hot spa (min)			
<10	1439	633	806
10–19	2931	1337	1594
20–29	1900	837	1063
≥30	660	284	376
Years of habitual hot spa bathing			
<10	759	377	382
10–19	1251	589	662
20–29	965	439	526
30–39	1037	469	568
≥40	2918	1217	1701
Time of hot spa bathing			
Before 9:00	971	463	508
9:00 to 13:00	803	350	453
13:00 to 19:00	2835	1333	1502
19:00 or after	2321	945	1376

The number of respondents with regard to three conditions (duration of immersion in hot

spa, years of habitual hot spa-bathing, and time of hot spa-bathing) are presented.

**Table 3:** Respondents’ use of respective hot spa quality types.

Hot spring type	Total	Men	Women
Total	3718	1679	2039
Simple	***2160***	***994***	***1166***
Chloride	***1093***	***496***	***597***
Bicarbonate	***328***	***134***	***194***
Sulfur	85	28	57
Iron	23	12	11
Sulfate	13	9	4
Carbon dioxide	14	6	8
Acid	1	0	1
Aluminum	1	0	1

Hot spa quality types with more than 100 users are shown in bold italic columns; these

were subjected to subsequent analyses.

First, we tried to detect differences in the disease incidence rate between spa bathers and non-spa bathers (Tables 4–5). Men and women were analyzed separately to avoid gender-associated confusion. Crude data seemed inaccurate to detect the effects of spa-bathing because some people may take a hot spa bath under inappropriate conditions, which could mitigate any disease-preventive effects. However, individual bathing conditions should still be taken into account when evaluating the disease-preventive effects of spa-bathing. Medical events occurring in the previous year can still be regarded as resulting from habitual bathing.

**Table 4:** Comparison of disease incidence in lifetime between hot spa bathers and non-hot spa bathers by gender.

Men						Lifetime	Women					
Non-spa bather	Spa-bather	Pre-adjusted		Adjusted			Non-spa bather	Spa-bather	Pre-adjusted		Adjusted	
**990**	**3091**	**OR**	**p-value**	**OR**	**p-value**	**Participants**	**1332**	**3839**	**OR**	**p-value**	**OR**	**p-value**
**155**	**540**	1.140	0.186	1.104	0.284	**Cancer**	**175**	**524**	1.045	0.638	1.020	0.817
**103**	**303**	0.936	0.582	0.679	**0**.**000**	**IHD**	**71**	**165**	0.798	0.120	0.839	0.172
**114**	**320**	0.887	0.302	0.810	**0**.**049**	**Arrhythmia**	**108**	**297**	0.950	0.664	1.168	0.160
**425**	**1204**	0.848	**0**.**026**	0.869	0.053	**Hypertension**	**552**	**1397**	0.808	**0**.**001**	0.767	**0**.**000**
**36**	**111**	0.987	0.947	0.908	0.599	**Apoplexy**	**29**	**65**	0.774	0.255	1.384	0.052
**85**	**259**	0.974	0.839	0.937	0.621	**Gout**	**7**	**29**	1.441	0.385	1.795	0.212
**36**	**112**	0.996	0.985	0.965	0.856	**Asthma**	**54**	**145**	0.929	0.651	0.783	0.098
**181**	**562**	0.993	0.943	0.922	0.386	**DM**	**137**	**373**	0.939	0.548	1.238	**0**.**039**
**120**	**274**	0.705	**0**.**003**	0.651	**0**.**000**	**Hyperlipidemia**	**193**	**488**	0.859	0.098	0.879	0.163
**46**	**143**	0.995	0.979	0.744	0.057	**Renal disease**	**43**	**92**	0.736	0.101	0.666	**0**.**022**
**24**	**40**	0.528	**0**.**013**	0.353	**0**.**000**	**Depression**	**43**	**80**	0.638	**0**.**018**	0.916	0.628
**25**	**61**	0.777	0.293	0.427	**0**.**000**	**Chr Hepatitis**	**23**	**72**	1.088	0.728	0.952	0.849
**13**	**46**	1.135	0.688	1.241	0.455	**Collagen Dis**	**39**	**129**	1.153	0.443	1.405	0.053
**42**	**124**	0.943	0.749	0.672	**0**.**035**	**Allergy**	**98**	**268**	0.945	0.644	0.881	0.299
**819**	**2528**	0.938	0.502	0.823	**0**.**016**	**Total Patients**	**1046**	**2868**	0.808	**0**.**005**	0.769	**0**.**000**

P-values with a significant difference between hot spa bathers and non-hot spa bathers are

shown in bold. IHD; ischemic heart disease; DM: diabetes mellitus; Chr Hepatitis: chronic

hepatitis; Collagen Dis: collagen disease.

**Table 5:** Comparison of disease incidence in the previous year between hot spa bathers and non-hot spa bathers by gender.

Men		Previous 1 year	Women	
Pre-adjusted	Adjusted		Pre-adjusted	Adjusted
0.570	0.748	Cancer	**0**.**028**	**0**.**001**
0.513	0.193	IHD	0.972	0.367
0.664	0.52	Arrhythmia	0.487	0.338
0.653	0.243	Hypertension	0.501	**0**.**000**
0.327	0.757	Apoplexy	0.307	0.517
0.231	0.454	Gout	0.972	0.287
0.396	0.127	Asthma	0.610	0.577
0.944	0.728	DM	0.555	0.646
0.824	0.709	Hyperlipidemia	0.421	**0**.**007**
0.972	0.283	Renal Disease	**0**.**016**	0.376
0.077	0.168	Depression	0.610	0.892
—	—	Chr Hepatitis	0.556	0.730
0.086	**0**.**000**	Collagen Dis	0.077	**0**.**022**
0.257	0.551	Allergy	0.174	0.098
0.861	0.848	Total	0.352	0.209

P-values with a significant difference between hot spa bathers and non-hot spa bathers are

shown in bold. IHD; ischemic heart disease; DM: diabetes mellitus; Chr Hepatitis: chronic

hepatitis; Collagen Dis: collagen disease.

The respondents were categorized according to spa-bathing conditions and medical history (previous year vs. lifetime; Supplemental Tables 1–8). Spa-bathing conditions with significant trends in terms of disease incidence are summarized in Supplemental Tables 9–24 and 6–9). The higher lifetime disease incidence rate observed in spa bathers suggests that spa-bathing has a promotive effect on disease incidence. However, the higher lifetime incidence rate of life-threatening diseases such as cancer should be interpreted differently. If a higher life-time cancer incidence of spa-bathers is observed, that means that there are more cancer survivers who recovered, take spa-bath habitually and send back a filled questionnaire to us. Such a finding suggests that more cancer patients could survive in spa-bathers than in non-spa-bathers, indicating an anti-cancer effect of habitual spa-bathing. The effects of spa-bathing on cancer (according to affected organ) are summarized in Tables 6–9.

**Table 6:** Correlation between disease incidence and hot spa-bathing conditions in men.

Men	Cancer		IHD		Arrhythmia		HT		Apoplexy		Gout		Br Asthm		DM		HL		Renal Dis		Depression		Chr Hep		Coll Dis		Allergy	
Men	Life	1 yr	Life	1 yr	Life	1 yr	Life	1 yr	Life	1 yr	Life	1 yr	Life	1 yr	Life	1 yr	Life	1 yr	Life	1 yr	Life	1 yr	Life	1 yr	Life	1 yr	Life	1 yr
1/W–M	−	+	−		−				−																			
2–3/W	+											+	−				−						−					
4–5/W	+	+	−				−		−														−					
1/Day			−				−		−								−		−		−		−			−		
<10 m	+		−		+												−		−		−		−			−		
10–19	+				−		−		−		−						−						−			−		
20–29			−	−		+		+												+	−					−		
≥30 m			−		−										−								−					
<10 yr	+		−																		−		−		+		−	
10–19					−							+					−						−					
20–29			−		−				−				−				−		−		−							
30–39			−			+		+					+		−					+							−	
≥40 yr				−	−		−				+		+				−				−		−			−		
Before 9																	−				−							
9–12	+																−											
13–18		−				+		+																				
19-				−			−		−								−				−							
Simple			−	−			−	+	−				−				−				−				+	−		
Chloride					−	+	−		−			+									−							
Bicarbon							−		−		−				−		−		−				−					

(+) Indicates that the disease incidence was higher in hot spa bathers than in non-hot spa bathers. (−) Indicates that the disease incidence was lower in hot spa bathers than in non-hot spa bathers.

IHD; Ischemic heart disease, HT; hypertension, Br Asthm; Bronchial asthma, DM; Diabetes Mellitus, HL; Hyperlipidemia, Renal Dis; Renal disease, Chr Hep; Chronic Hepatitis, Collagen Dis; Collagen Disease. Ur Bladder; Urinary Bladder, Leuk/Lym; Leukemia/Lymphoma.

**Table 7:** Correlation between disease incidence and hot spa-bathing conditions in women.

Women	Cancer		IHD		Arrhythmia		HT		Apo		Gout		Br Asthm		DM		HL		Renal Dis		Depression		Chr Hep		Coll Ds		Allergy	
Men	Life	1 yr	Life	1 yr	Life	1 yr	Life	1 yr	Life	1 yr	Life	1 yr	Life	1 yr	Life	1 yr	Life	1 yr	Life	1 yr	Life	1 yr	Life	1 yr	Life	1 yr	Life	1 yr
1/W–M																												
2–3/W								−	+				−		+										+	+		
4–5/W			−		+		−	−																		+		
1/Day	−	−	−				−		−												−							
<10 m	−	−			+		−	−	+		+				+						−					+	−	
10–19	−	−			+		−		−				−		+	+	−	−					+		+		−	
20–29	+		−		+		−	−	+									−	−						+	+	+	
≥30 m					−		−										−											
<10 yr	−	−					−	−					−		+			−	−							+		
10–19		−						−						+											+			
20–29		−	−				−		−										−		−				+	+		
30–39	+				+		−				+				+													
≥40 yr		−	+		+		−	−	−								−				−						−	
Before 9	−						−		−										+				+		+	+		
9–12								−	+				−		+				−							+		
13–18		−					−	−													−							
19−		−	−				−														−				+	+		
Simple					+		−								+						−				+	+		
Chloride		−			−		−																			+		
Bicarbon	−						−						+				−

**Table 8:** Correlation between cancer incidence and hot spa–bathing conditions in men.

Men	Stomach		Colon		Pancreas		Liver		Breast		Lung		Larynx		Thyroid		Skin		Kidney		Ur Bladder		Prostate		Brain		Leuk/Lym		Bone	
	Life	1 yr	Life	1 yr	Life	1 yr	Life	1 yr	Life	1 yr	Life	1 yr	Life	1 yr	Life	1 yr	Life	1 yr	Life	1 yr	Life	1 yr	Life	1 yr	Life	1 yr	Life	1 yr	Life	1 yr
1/W–M					+	+												+			+									
2–3/W	+						+	+																						
4–5/W	+			−													+	+					+							
1/Day				−															−											
<10 m	+						+	+										+	−		+									
10–19	+			−	+	+					+		+				+		+				+							
20–29	+		+	−			+												−				−							
≥30 m																														
<10 yr	+						+											+												
10–19					+	+					+		+				+		+		+		+							
20–29							+	+																						
30–39	+		+															+			−		−							
≥40 yr				−							+		+					+									−			
Before 9																														
9–12	+				+		+	+										+												
13–18	+			−																										
19−				−																+					+					
Simple	+			−			+						+								+		+							
Chloride																					+									
Bicarbon	+		+	+																										

(+) Indicates that the cancer incidence was higher in hot spa bathers than in non–hot spa bathers. (−) Indicates that the cancer incidence (c, d) was lower in hot spa bathers than in non-hot spa bathers.

**Table 9:** Correlation between cancer incidence and hot spa-bathing conditions in women.

Women	Stomach		Colon		Pancreas		Liver		Breast		Uterine		Ovary		Lung		Larynx		Thyroid		Skin		Kidney		Ur Bladder		Brain		Leuk/Lym		Bone	
	Life	1 yr	Life	1 yr	Life	1 yr	Life	1 yr	Life	1 yr	Life	1 yr	Life	1 yr	Life	1 yr	Life	1 yr	Life	1 yr	Life	1 yr	Life	1 yr	Life	1 yr	Life	1 yr	Life	1 yr	Life	1 yr
1/W–M															+																	
2–3/W	+	+													+				+													
4–5/W											−																+					
1/Day	−							−−																	+							
<10 m													+						−													
10–19			−						−						+							−										
20–29		+													+				+								+		−			
≥30 m															+				+													
< 10 yr			−						−										+										−			
10–19									−						+		+														+	
20–29	−								−						+																	
30–39	+	+							+						+								+				+					
≥40 yr			+					−																								
Before 9																																
9–12		+													+				+													
13–18								−							+	−						−										
19−							+		−		+				+		+															
Simple		+																	+													
Chloride													+																			
Bicarbon																							+									

### Trends associated with spa-bathing in individual disease categories

The preventive and promotive effects of spa-bathing on disease were estimated by analyzing a combination of disease incidence rates (lifetime and previous year) and spa-bathing conditions (Tables 6–9). A higher or lower lifetime incidence of a condition indicated that spa-bathing prevented or promoted the disease.

Cases in which either a higher or lower incidence of the same disease was observed with different spa-bathing conditions, or in which there was a discrepancy between the lifetime and previous year incidence trends, should be carefully interpreted.

In men, a discrepancy was observed in arrhythmia, hypertension, renal disease, gout, and collagen disease. Arrhythmia, hypertension, and renal disease showed a lower lifetime incidence, but a higher incidence in the previous year among those who bathed for a duration of 20–29 min and those who had 30–39-years of habitual hot spa-bathing. In such a mixing finding, the occurrence of a disease is suppressed during a period of its lower incidence, when the number of people without this disease is accumulated. After the suppression period, the disease occurs among the accumulated population. As a result, higher incidence of the disease will be observed. So, the observed results suggest that 29–38 years of habitual spa-bathing delays the onset of disease, and that the preventive effects of spa-bathing are most consistently achieved by a bathing duration of 20–29 min. Arrhythmia, hypertension, and renal disease are classified as cardiovascular diseases, and thus the lower blood pressure associated with these conditions likely contributed to this result. In men, a higher incidence of gout was observed in those who had over 40 years of habitual-spa-bathing, implying that spa-bathing had no preventive effects. However, a bathing duration of 10–19 min in a bicarbonate spa may have some preventive effects. In addition, among men, some bathing conditions were associated with a higher incidence of collagen disease, but no bathing conditions were associated with a lower incidence, indicating that spa-bathing has promotive effects on this disease. In men, spa-bathing also seemed to have preventive effects for non-cancer diseases, except for gout and collagen disease.

In women, many bathing conditions were associated with a lower incidence of cancer. A lower incidence rate in the previous year was observed in those who had less than 30 years of habitual spa-bathing, and a higher lifetime incidence rate was seen in those who had 30–39 years of habitual spa-bathing, indicating that spa-bathing may delay cancer onset by 30 years.

The results showed that spa-bathing may also prevent ischemic heart disease, hypertension, bronchial asthma, hyperlipidemia, renal disease, depression, and allergies in women because the lifetime incidence rates of these diseases were lower under many bathing conditions. However, the results showed that spa-bathing may also promote arrhythmia, diabetes mellitus, collagen disease, gout and chronic hepatitis, even though the associated bathing conditions were inadequate to confirm the effects. Moreover, the effect of spa-bathing on apoplexy was unclear because of mixed results regarding the disease incidence.

Cancer is the leading cause of death among Japanese people. Most cancer patients are expected to die within several years after diagnosis. A bathing condition associated with a higher lifetime incidence of cancer can be derived from an increased number of long-term survivors (Tables 6–9). If a higher incidence rate in the previous year and a lower or stable lifetime incidence rate are observed, then cancer incidence has been suppressed until one year previously.

Regarding the incidence of cancer in relation to specific organs among men, the effects of spa-bathing were unclear for breast, thyroid, and bone cancer, as well as leukemia/lymphoma and brain tumors, because no or only one hot spa-bathing condition was associated with a significant incidence rate. Reliable candidates for bathing conditions with preventive effects on cancer incidence were considered to involve three or more cases. A higher incidence of cancer in the previous year was observed among bathers for the pancreas, liver, and skin. Regarding the years of habitual bathing, preventive effects were seen for 9–18 years in pancreas cancer, 19–28 years in liver cancer, and an inconsistent period in skin cancer. No effects of spa-bathing on cancer incidence could be identified in cases for which no significant trends in cancer incidence during the previous year were seen among spa bathers. Even in this case, a higher lifetime incidence among spa bathers implied that spa-bathing could have preventive effects on cancer and increase survival by suppressing cancer recurrence. These cases involved gastric, colon, liver, lung, laryngeal and skin cancer. However, because of mixed results regarding lifetime incidence, whether hot spa-bathing had preventive or promotive effects on urinary tract cancer (kidney, urinary bladder, and prostate) in males was unclear. Regarding colon cancer in men, a reduced incidence in the previous year and an increased lifetime incidence under some bathing conditions clearly suggested that spa-bathing can suppress the occurrence of colon cancer.

In women, the incidence of gastric, colon, liver, breast, lung, thyroid, and brain cancer showed significant associations with three or more bathing conditions. In the cases of gastric, colon, and breast cancer, the lifetime incidence rate seemed to increase after a year of bathing, indicating that cancer incidence was suppressed and delayed. In regard to lung cancer, the incidence was increased under some bathing conditions, but no conditions were associated with a decrease in lifetime incidence or an increase in the incidence during the previous year, indicating an accumulation of lung cancer survivors. This finding was supported by the increased incidence observed in those who had 10–39 years of habitual spa-bathing. Regarding liver cancer, a lower incidence rate was observed in long-term daily bathers, but the effect on prolongation of cancer survival was unclear. Regarding thyroid cancer, a higher incidence was observed only in those who had less than 10 years of habitual spa-bathing, and no changes in incidence were seen in other periods, indicating that there was no improvement in cancer survival for those who had more than 10 years of habitual spa-bathing. This implies that spa-bathing may advance and promote the onset of thyroid cancer. However, the results also showed that 10 minutes or less of spa-bathing may prevent thyroid cancer. Regarding brain tumors among women, the lifetime incidence rate increased among those with 30–39-years of habitual spa-bathing, indicating prolonged survival.

## Discussion

The present study found that habitual spa-bathing can affect the incidence rate of various diseases. Spa-bathing time, which is defined by three factors (frequency, soaking time, and years of habitual bathing), did not show a simple linear correlation with the overall disease incidence rate. Spa-bathing does not always prevent disease occurrence, and may actually promote disease occurrence under certain conditions. The use of hot spa-bathing therefore for health promotion depends on a combination of spa-bathing conditions, individual physical characteristics, and lifestyle.

Previous studies have reported that spa-bathing exerts a wide range of therapeutic effects. However, these effects have only been reported for patients, not healthy people. Some of possible preventive effects of habitual spa-bathing in this study could or could not be extrapolated from previous observations of patients. Spa-bathing-associated preventive effects for hypertension, hyperlipidemia, and depression could be extrapolated from previous reports^2–4^. In addition, the preventive effects for hypertension may reduce the occurrence of arrhythmia, apoplexy, ischemic heart disease, and renal disease.

Immunopotentiation by heat has also been reported, which supports the associations observed between spa-bathing and both increased cancer-survival and the occurrence of autoimmune diseases, including collagen disease^5–7^. Moreover, such a systemic immunopotentiation may change the constitution of intestinal floral, which can contribute to the suppression of colon carcinogenesis^8^.

Diabetes mellitus among women could not always be extrapolated from previous reports. Hot spa therapy can be effectively used to reduce blood glucose level^9^. However, spa-bathing has been shown to increase blood retention in the bowels because of dilated intestinal blood vessels and decreased blood circulation in the portal vein^10^. These effects may influence the distribution of absorbed nutrients, which would be followed by high blood sugar, possibly leading to impaired glucose tolerance^11^. The reported effects of spa-bathing on blood glucose are not consistent. This may be because the results depend on the spa-bathing conditions. A promotive effect of spa-bathing on diabetes mellitus was observed only in women. Body-warming by spa-bathing may lead to the increased circulation of heat shock proteins. The serum level of Hsp-70 only increases among women with diabetes mellitus^12^, and plays a causative role in gestational hyperglycemia^13,14^. Therefore, body-warming can cause specific responses involving heat shock proteins in women. In addition, spa-bathing lowers portal venous pressure, leading to the prevention of hepatitis, which supports the lower incidence of chronic hepatitis seen among spa bathers. Further accumulation of new discoveries about the biological effects of body-warming is need to clarify the underlying mechanisms for some correlations between past disease incidence and habitual spa-bathing observed in the present study; these could not be extrapolated in previous observations. Body-warming should be expected to increase the expression of heat shock proteins from somatic cells. Generally, heat shock proteins serve as molecular chaperones that allow cells to adapt to gradual environmental changes and to survive under otherwise lethal conditions^15^, leading to the suppression of oncogenesis and thereby supporting our observation of more cancer survivors among spa bathers. Heat shock proteins play an important role in the suppression of cardiovascular complications associated with hyperglycemia in diabetes mellitus^16^. These observations support our finding that cardiovascular diseases were suppressed in spa bathers; however, this cannot be extrapolated to diabetes mellitus. Continuous mild systemic heat stress may suppress the cellular degeneration of beta islet cells, but under female-specific endocrinological conditions, body-warming can also induce glucose intolerance, as described above.

Even in the early stage of thermal medicine, the correlation between habitual spa-bathing and disease incidence presented here could be expected to serve as a basic guide for healthcare in relation to hot spa-bathing.

An epidemiological study such as the present study can only be conducted in a country such as Japan, where people take a bath daily. Although this study was based on data from a limited area, the results provide some evidence that body-warming yields positive results in preventive medicine, and this could represent the beginning of ‘thermal preventive medicine’. In the present study, easily answerable questions were selected, and all questions were designed for seniors. Therefore, we could only adopt items associated with spa-bathing; questions in relation to the occurrence of lifestyle-related disease, including habitual exercise, smoking, alcohol intake, body mass index, family members, educational status, and economical condition, were omitted. These conditions were considered similar between spa bathers and non-spa bathers in this study. Moreover, we did not record the temperature of hot spa baths because Japanese people have no custom of checking the water temperature. These issues should be addressed in future research with a satisfactory number of responses.

In Japan, where the aging of the population has been rapidly accelerating, the expansion of medical expenses for the elderly is a major socioeconomic problem. Utilizing hot spas to help prevent disease could be one way to help deal with this problem. Along with the aging of the population and the development of new medical technologies, it is important to periodically review the medical effects of hot spas. It is also important to repeatedly conduct surveys that include both hot spa-bathing conditions and other conditions associated with lifestyle.

Beppu citizens did not show preferable results over the general Japanese population in the occurrence of disease or life expectancy^17^. In our analysis, the significant effects of hot spa-bathing were hidden in many bathing conditions before adjustment. According to a report from Beppu city, the mortality rate of colon cancer in Beppu city is similar to that of the general population, contrary to our observations. Beppu citizens have not always used hot spa-bathing in an appropriate way for good health. Certain bathing conditions are preventive for some diseases, but promotive for others. This makes it difficult for each person to find the most suitable way to prevent disease. Even so, the conclusive findings describing the positive or negative correlations between spa-bathing conditions and the occurrence of various diseases suggests suitable bathing conditions to prevent and avoid the occurrence of numerous diseases. We hope that the results of this study represent a first step toward the expanded use of hot spa-bathing for the elongation of healthy life expectancy. Furthermore, we hope these findings can provide a preventive medicine framework for other countries to take advantage of daily body warming.

## Methods

### Questionnaire survey

**Figure 1:**
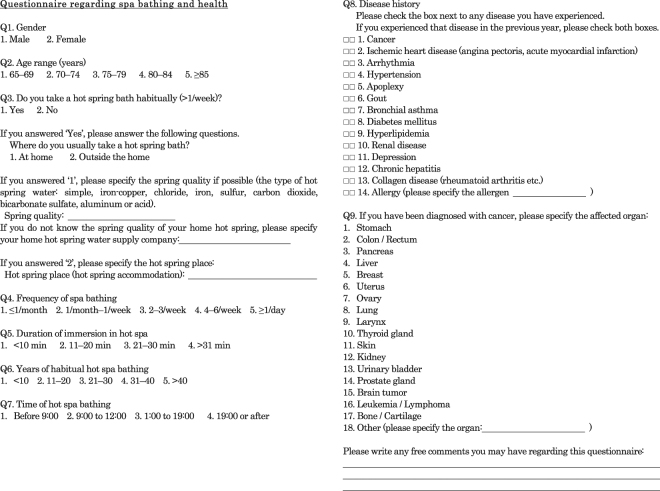
The English version of the Japanese questionnaire used in the present study.

As of 2011, there were 34,465 citizens aged ≥ 65 years living in Beppu city. Questionnaires regarding hot spa-bathing habits and disease history were randomly sent to 20,000 of these residents (Fig. 1). Questions concerning the companies that supplied hot spa water to their home were included to identify water quality types. We received 11,146 responses, 9,252 (4,081 men, 5,171 women) of which were valid and subsequently analyzed. Spa water quality type was identified in 3,843 of the responses. The questionnaire also asked about their personal medical histories in 14 disease categories and 17 cancer types, including whether they had experienced any diseases with in the previous year. The present research was performed, following the approval by the Conjoint Health Research Ethics Board of Kyushu University.

## Statistical analysis

In this survey, those responding that they took a hot spa bath more than once a month and once a month or less (least frequent spa-bathers), were defined as hot spa bathers (spa bathers) and non-hot spa bathers (non-spa bathers), respectively. First, diseases occurrence rates under four bathing conditions (frequency, immersion time, length of habitual bathing in years, and time zone) were compared between spa and non-spa bathers. When a previous disease occurred with one of the four conditions analyzed, the other three conditions were adjusted. If the crude values before adjustment were used, this is indicated as “pre-adjusted” in the Figures and Tables. In the analysis of the effects specific for each hot spa type, all four conditions were adjusted. Odds ratios were calculated for the incidence of each previous disease and compared between spa bathers and non-spa bathers using the chi square test. In cases for which the 95% confidence interval did not contain the value of 1.0 (p < 0.05), the difference was regarded as statistically significant.

## Electronic supplementary material

Supplmentary Table 1-24
